# CuidaCare: effectiveness of a nursing intervention on the quality of life’s caregiver: cluster-randomized clinical trial

**DOI:** 10.1186/1472-6955-13-2

**Published:** 2014-01-27

**Authors:** Milagros Rico-Blázquez, Esperanza Escortell-Mayor, Isabel del-Cura-González, Teresa Sanz-Cuesta, Pilar Gallego-Berciano, Gonzalo de las Casas-Cámara, Sonia Soto-Díaz, Petra García-Sanz, Natalie Harris-de-la-Vega, María Martín-Martín, Lorena Domínguez-Pérez, Araceli Rivera-Álvarez, Juan Antonio Sarrión-Bravo, Concepción Pérez-de-Hita, Marisol de-Frías-Redondo, Carmen Ferrer-Arnedo, Montserrat Hernández-Pascual, Antonio Valdivia-Pérez, Yolanda Ramallo Fariña

**Affiliations:** 1 Unidad de Apoyo a la Investigación. Dirección Técnica de Docencia e Investigación. Gerencia Adjunta de Planificación y Calidad. Gerencia de Atención Primaria, C/ San Martín de Porres, 6 _ 5ª planta, 28035 Madrid, Spain; 2 Investigadora de la Red de Investigación en Servicios Sanitarios de Salud y en Enfermedades Crónicas (REDISSEC), Madrid, Spain; 3 Facultad de Enfermería, Universidad de Alcalá, Alcalá de Henares, Spain; 4 Facultad de Ciencias de la Salud, Universidad Rey Juan Carlos, Madrid, Spain; 5 Unidad Docente de Medicina Preventiva y Salud Pública. Comunidad de Madrid, Hospital Universitario de La Princesa, Diego de León, 62, Madrid 28006 Spain; 6 FEA Medicina Preventiva y Salud Pública, Hospital Universitario General de Castellón, Av Benicasim, Castellón de la Plana 12004 Spain; 7 Unidad Docente Multiprofesional Oeste, Servicio Madrileño de Salud, Calle Alonso Cano, 8, Mostoles 28933, Spain; 8 Centro de Salud Juncal, Servicio Madrileño de Salud, Avda. Madrid /Esq. Brasil, 39, Torrejón de Ardoz 28850, Spain; 9 Centro de Salud La Veredilla, Servicio Madrileño de Salud, C/ Turin, 37, Torrejón de Ardoz 28850, Spain; 10 Centro de Salud Miguel de Cervantes, Servicio Madrileño de Salud, Avda. Gustavo Adolfo Bécquer n°23, Alcalá de Henares 28, Spain; 11 Dirección Asistencial Este Atención Primaria, Servicio Madrileño de Salud, C/ Nuestra Señora del Pilar, s/n, Alcalá de Henares 28803, Spain; 12 Gerencia Hospital de Guadarrama, Servicio Madrileño de Salud, Avda. del Molino del Rey, 2, Guadarrama 28440, Spain; 13 Dirección Técnica de Sistemas de Información Sanitaria de la Gerencia de Atención Primaria, Servicio Madrileño de Salud Salud, Calle O’Donnell 55, Madrid 28009, Spain; 14 Unidad de Medicina Preventiva, Hospital de Dénia-Marina Salud, Ptda de Beniadlá s/n, Dénia 03700, Spain; 15 FUNCIS: Fundación Canaria de Investigación y Salud, (Camino Candelaria, 44), Tenerife, (38109), Spain. Centro de Investigaciones Biomédicas de Canarias (CIBICAN), Universidad de La Laguna, La Laguna, Spain

**Keywords:** Caregivers, Nursing, Primary care, Quality of life, Social support, Psychological adaptation, Caregiver burden

## Abstract

**Background:**

In Spain, family is the main source of care for dependent people. Numerous studies suggest that providing informal (unpaid) care during a prolonged period of time results in a morbidity-generating burden. Caregivers constitute a high-risk group that experiences elevated stress levels, which reduce their quality of life.

Different strategies have been proposed to improve management of this phenomenon in order to minimize its impact, but definitive conclusions regarding their effectiveness are lacking.

**Methods/Design:**

A community clinical trial is proposed, with a 1-year follow-up period, that is multicentric, controlled, parallel, and with randomized allocation of clusters in 20 health care centers within the Community of Madrid. The study's objective is to evaluate the effectiveness of a standard care intervention in primary health care (intervention CuidaCare) to improve the quality of life of the caregivers, measured at 0, 6, and 12 months after the intervention.

One hundred and forty two subjects (71 from each group) ≥65 years, identified by the nurse as the main caregivers, and who provide consent to participate in the study will be included.

The main outcome variable will be perceived quality of life as measured by the Visual Analogue Scale (VAS) of EuroQol-5D (EQ-5D). The secondary outcome variables will be EQ-5D Dimensions, EQ-5D Index, nursing diagnosis, and Zarit's test. Prognostic variables will be recorded for the dependent patient and the caregiver.

The principle analysis will be done by comparing the average change in EQ-5D VAS value before and after intervention between the two groups. All statistical tests will be performed as intention-to-treat. Prognostic factors' estimates will be adjusted by mixed-effects regression models. Possible confounding or effect-modifying factors will be taken into account.

**Discussion:**

Assistance for the caregiver should be integrated into primary care services. In order to do so, incorporating standard, effective interventions with relevant outcome variables such as quality of life is necessary. Community care nurses are at a privileged position to develop interventions like the proposed one.

**Trial registration:**

This trial has been registered in ClinicalTrials.gov under code number NCT 01478295.

## Background

### Aging and dependency

Population aging constitutes one of the most relevant demographic phenomena of this century. The *World Population Ageing (2009)* report published by the United Nations in January 2010 shows that we are facing a general and unprecedented problem. According to their data and estimations, 11% of the population was >60 years old in 2009, and this figure is estimated to increase up to 22% in 2050. The report highlights that one of the fastest growing segments of the population will be the >80 year old, which will constitute 20% of the >60 year old group by 2050 [1].

The situation in Spain shows similar figures. The current aging index is 17%, 4% of people are >80 years old, and it is forecasted that 37% of the total population will be ≥60 years by 2049 [2].

This demographic phenomenon poses important economic and social health-related challenges. In the social realm, population aging influences migration tendencies as well as family composition and individual roles within it. In the health setting, morbidity related to the aging process and increases in the prevalence of chronic and degenerative diseases entails the emergence of dependency and a growing need for health care and services [3].

The European Council defines dependency as the state in which people, due to causes linked to the lack or loss of physical, psychological, or intellectual autonomy, are in need of assistance and/or significant help to carry out common activities of daily life, and particularly those related to personal care [4].

In Spain it is estimated that 9% of the total population present some handicap or limitation, of which one third shows severe dependency requiring help from someone to perform basic activities of daily living (BADL) [5]. The percentages of people >65 and >85 years old with any handicap are 32.2% and 63.3%, respectively. According to the *Encuesta Nacional de Salud 2011/2012*, 34.2% of the population ≥65 years in Spain state being in need of help for carrying out any BADL related to personal care, 37.5% for performing house work, and 36.2% do not consider themselves able to perform those related to mobility [6].

### Care of the dependent person

In situations of dependency, two care alternatives are usually present, formal and informal. Formal care has been defined as those services that are offered by a professional in a specialized manner and go further than the capabilities that people have to take care of themselves or others. It is usually provided by public institutions or hired from private companies by the family [7].

Informal care is that which stems from the family environment or social network of the dependent person. Its main features are its unpaid character, being carried out in the private realm, having a highly marked domestic content, and a mainly female profile. The person who assumes responsibility for the support and daily care of the dependent person is called the main caregiver [8].

There is interdependency between the formal and informal care sectors. In Spain, our model of home assistance for dependent people is based on the family as the care-giving unit, and health care is mainly provided by primary care. It is estimated that the family bears 88% of the total care time consumed by the dependent person [9]. The typical profile of the main caregiver is female (83%), of low educational level, who invests an average of 4 hours a day, without rest breaks, and receiving scarce institutional support [7]. According to the *Encuesta Nacional de Salud 2011/2012 *[6], 16.6% of men and 49.4% of women who live with handicapped people are responsible for providing care to them all by themselves.

### The main caregivers as a vulnerable group

Providing informal care is considered as an important stress source, even resulting in higher stress levels for caregivers than for the patients themselves [10].

In the last years, numerous articles have concluded that providing informal care constitutes an under-diagnosed, morbidity-generating circumstance [11-13]. The outcome of a prospective cohort study with a 4.5-year follow-up period indicated that married caregivers between 66 and 96 years old who took care of their couples had a 63% higher probability of dying from any cause within a follow-up period of 4 years compared to a control group of non-caregivers [12].

Studies of qualitative outcomes, such as the one by Teschendorf et al. [13], identify 4 categories of concerns and requests of cancer patients' caregivers: health care, strategies about how to deal with the role of a caregiver, emotional support, and social recognition.

### Interventions and strategies that take care of the main caregiver

In the last years, there is remarkable interest in evaluating the effect that interventions for the caregiver have on their health condition and quality of life, and published relevant systematic reviews are plentiful.

In a systematic review including 112 clinical trials whose objective was to evaluate the effect of different interventions on the burden of the main caregiver of demented patients, Goy et al. [14] conclude that complex interventions reduce the burden and increase the capacity to deal with it, especially when they are personalized, provided in the home, and oriented towards the specific needs of the caregiver-patient pair. The effect of personal assessment and support groups may vary depending on the trainer’s degree of skill, environment, frequency and duration of the contact, and employed teaching materials.

In another systematic review published by Parker et al. [15] to evaluate the effectiveness of psycho-educational interventions for caregivers of non-institutionalized patients with dementia, 12 of the 13 reviewed studies observed a significant association between such interventions and caregivers’ depression and overload. These authors conclude that interventions are more effective if there is a personalized program, active participation of the caregiver, and specific information is given. Those interventions that only provide mutual support, group work, and self-help materials do not seem to affect the burden.

In 2011, The Cochrane Collaboration published 2 systematic reviews [16,17] that evaluated the effectiveness of support interventions on the health of main caregivers of dependent people suffering from different pathologies.

Candy et al. [16] included 11 randomized controlled trials with 1,836 caregivers of terminal stage patients who received support interventions for the caring of the dependent person, emotional support, and/or tools to deal with it. The results suggest that emotional support interventions may help reduce the psychological unrest of the caregivers.

Legg et al. [17] included 8 clinical trials with a total of 1,007 caregivers of dependent patients suffering from stroke sequelae. They evaluated the effect of non-pharmacological interventions versus standard care. They classified the interventions into support and information, teaching of procedures, and psycho-educational. The results suggest that psycho-educational interventions before discharge of the patient have greater impact on the caregiver's burden.

However, it is difficult to draw conclusions about the effect that the interventions directed at the main caregiver have on their health and quality of life. The main limitations described in these reviews are related to the large number of different interventions evaluated, their complexity, and the difficulty of reproducing them [14-17].

Among the recommendations for future research, it is suggested that studies evaluate interventions in various contexts, implement methods to avoid losses to follow-up of the studied subjects, and evaluate different implementation strategies to determine the interventions’ acceptability, viability, efficacy, and cost-effectiveness [14].

Review studies show that quality of life, anxiety and depression levels, stress, and overload of the main caregiver are the outcome measurements used most frequently as main or secondary variables to evaluate the effectiveness of interventions aimed at the caregiver [16,17].

Several studies from our setting use EuroQol (EQ-5D) [18,19]. This tool has advantages in the setting of our study since it is a culturally-adapted instrument, highly validated in the Spanish population, with easy and rapid implementation, and which counts on standard scores obtained from the general population that can be used as reference values to compare against those obtained from studied patients and thus define the effect of the disease on the health-related quality of life (HRQL) [20,21].

### Structured care intervention in the area of primary care: intervention CuidaCare

The *Estrategia para el Abordaje de la Cronicidad en el Sistema Nacional de Salud *[22] considers the home as the ideal place for the care of dependent patients, since staying in one's own environment improves well-being and quality of life. It recognizes nurses, within a multidisciplinary team, as the professionals who lead and coordinate health care actions, both for the dependent person who requires home care and for their main caregiver. For the development of support and care programs aimed at chronic patients, the strategy indicates that they must be limited to not only developing informative activities and training on caregiving for the dependent person, but also include interventions for the caregivers themselves, addressing their problems and preventing pathological conditions and the risk of giving up.

In the Community of Madrid, the *Cartera de Servicios estandarizados de Atención Primaria *[23] considers the care of dependent patients within their scope of services, but does not explicitly consider the care of the main caregiver.

Normalization of nursing diagnostic process and usage of internationally recognized taxonomies (such as the NANDA-I for formulation of care problems, the NIC for intervention definition, and the NOC for result measurement) reduce variability in nursing clinical practice and facilitate normalization of caregiving, in addition to providing a tool that enables carrying out studies on the effectiveness of the interventions [24,25].

As a result, it seems adequate to put into practice community trials that allow for evaluating the effectiveness of complex, structured, and personalized nursing interventions.

The CuidaCare project proposes a community, pragmatic trial in primary care to evaluate the effectiveness of a complex, standard nursing intervention performed at the caregiver’s home that will measure relevant outcomes of the main caregivers.

### Hypothesis

A standard care intervention (intervention CuidaCare) in primary care is more effective than standard practice for improving the quality of life of old caregivers, as measured by the EQ-5D VAS, increasing the average pre- and post-intervention score change by ≥15 points between the intervention and control groups.

### Objectives

The objective of this study is to evaluate the effectiveness of a standard caregiving intervention (intervention CuidaCare) in primary care to improve the quality of life of main caregivers >65 years, as measured by the EQ-5D VAS during follow-up visits at 0, 6, and 12 months after the intervention.

#### Secondary objectives

• Describe the socio-demographic profile of the dependent patient and the main caregiver.

• Describe the degree of burden for the main caregivers, measured with the Zarit's test.

• Compare the effectiveness of the CuidaCare intervention against the standard nursing practice for improving the caregiver’s quality of life, measured with the EQ-5D Dimensions and the EQ-5D Index, at the end of the intervention and during follow-up visits at 6 and 12 months.

• Compare the effectiveness of the CuidaCare intervention with standard nursing practice in reducing caregiver burden, as measured by nursing outcome NOC Indicators and Zarit's test at the end of the intervention and during follow-up visits at 6 and 12 months.

• Analyze socio-demographic factors, characteristics of the dependent patient, caregiving attributes, and formal support that characterize the quality of life of the main caregiver.

## Methods/Design

### Study design

A community, controlled, multicentric clinical trial with randomized allocation by clusters and a 1-year follow-up period.

The intervention will be carried out by voluntary nurses with at least 3 years of experience in primary care, from 20 primary health care centers (PHCC) in the Community of Madrid (Spain).

The randomized unit will be the PHCCs (clusters). The analysis unit will be the caregiver (check-list cluster CONSORT in Additional file 1).

### Studied subjects

Subjects ≥65 years, identified by the nurse as the main caregiver of a patient included in the home consultation service of the *Cartera de Servicios estandarizados de Atención Primaria *[23] at the moment of inclusion in the study.

The person who assumes responsibility for the support and daily care of the dependent patient is termed the main caregiver [8].

1. Inclusion criteria:

• Age ≥65 years.

• Caregivers who have been carrying out this function for >1 month.

• Time employed as main caregiver is >6 months per year (even during non consecutive months).

• Not currently under another therapeutic intervention to ease caregiver tension.

• Able to follow the specific requirements of the trial.

• Willing to participate in the trial and provide written consent.

2. Exclusion criteria:

• Main caregivers of institutionalized patients.

### Sample size

Sample size has been calculated considering a relevant post-intervention average difference of 15 points between the intervention and control groups measured on the EQ-5D VAS, and a standard deviation of 25 (estimation obtained from Roset et al. [26]). This difference is equivalent to an effect size of 0.6.

Assuming an alpha error of 0.05 and a beta error of 20%, 44 caregivers are necessary for each group.

Since randomization is done by clusters, the sample size is adjusted in order to take into account the design effect. We have considered an intraclass correlation coefficient of 0.02 [27] and assumed an average cluster sample size of 15 caregivers. The design effect is 1.28. With these assumptions and expecting a 20% loss to follow-up after a 1-year period, the required sample size is 142 (71 caregivers per group).

### Randomization

#### *Allocation unit*

Allocation will be done by clusters, and the randomizing unit will be the PHCCs.

#### *Sequence generation*

The 20 PHCCs will be allocated into intervention or control groups by using a stratified randomizing sequence by number of caregivers, generated by a computer (EPIDAT 3.1).

#### *Concealment of allocation*

Randomization will be done in a centralized way by a researcher who does not participate in the study and is blind to the PHCC identity.

Main caregivers will be consecutively included in the study. During the home visit to the dependent patient, those caregivers meeting inclusion requirements for the trial will be informed of it and offered participation. If accepting to participate, they will be asked to fill in and sign the written consent and the meeting of all inclusion criteria, and no exclusion criteria, will be verified.

Randomization of PHCCs will be done once the caregivers eligible for the study have been chosen, so that recruitment by health professionals is not influenced by their allocation to a study group [28,29].

### Masking

It is not possible to mask the intervention in this type of trials. Post-intervention variables measurement will be performed by a group of nurses who will not know which group the caregiver belongs to. In the same way, the analysis will be done by professionals unaware of the allocation.

### The intervention

#### *Control group*

Standard clinical practice: the home consultation service to immobile patients from the *Cartera de Servicios estandarizados de Atención Primaria *[23] defines the care to be provided to dependent patients. For the caregiver, there is no formal care and their care needs are addressed individually.

#### *Intervention group*

Dependent patients will be given the care defined in the home consultation service to immobile patients from the *Cartera de Servicios estandarizados de Atención Primaria *[23].

In addition to assisting the caregivers in their eventual needs for care, they will be provided with the assistance defined in the CuidaCare intervention.

CuidaCare is a complex intervention that consists of the combination of several components aimed at improving the quality of life of caregivers. It has been designed by a multidisciplinary group of primary health care professionals, experts at nursing care and psychological approach to behavior.

It uses strategies to improve dealing with the role of a caregiver, training on health-related self-care and dependent patients' care, as well as emotional support.

As methodological support for its development, the recommendations of the NANDA-I have been used, which propose outcome criteria (NOC) and nursing interventions (NIC) to be employed in order to facilitate research approach for studying the main nursing diagnosis for main caregivers.

**Figure 1:**
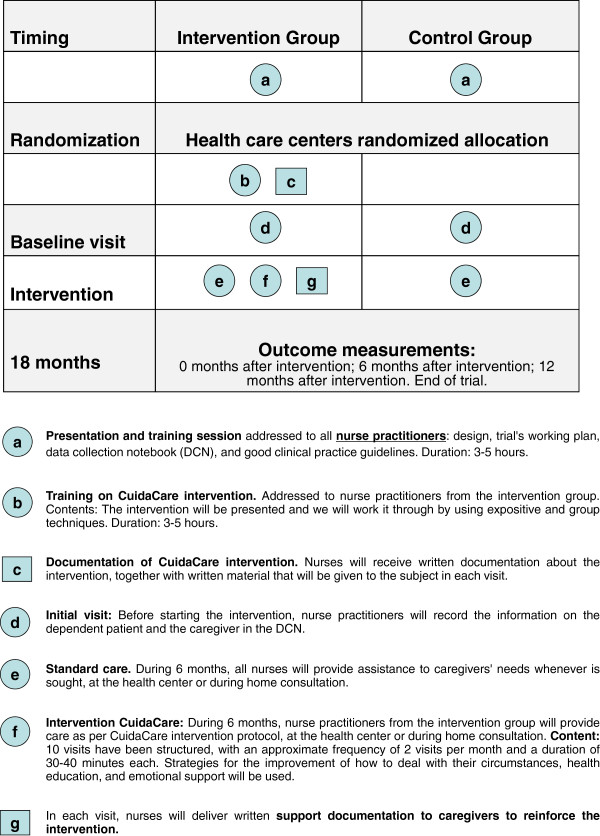
Componets of CuidaCare complex intervention vs standard clinical practice.

Figure 1 shows schematically the different components of both the control and the CuidaCare interventions [30].

Along the 10 programmed visits of the CuidaCare intervention, nurses allocated to this group will deal with the following content: health care system guidelines and caregiver support, nervousness and stress control, caregiver's self-care, self-esteem, self-learning, identifying sources of stress, dealing with problems, and improving communication skills.

### Variables

#### *Outcome variables*

The main outcome variable is quality of life perceived by the caregiver, expressed on the EQ-5D VAS [20,21]. This questionnaire comprises two sections: the VAS and the descriptive system EQ-5D Dimensions. In the VAS, subjects evaluate their own health condition on a scale ranging from 0 (worst imaginable health condition) to 100 (best imaginable health condition). The EQ-5D Dimensions system contains 5 questions where 5 dimensions of HRQL (Mobility, Self-Care, Usual Activities, Pain/Discomfort, Anxiety/Depression) are evaluated. Each question has 3 possible answers ranging from 1 (I do not have difficulties) to 3 (I have many difficulties).

#### *Health professional variables*

• Socio-demographic: sex and age.

• Years of experience in primary care and specific training in providing assistance to caregivers.

#### *Dependent patient variables*

• Socio-demographic: sex, age, marital status, and educational level.

• Degree of dependency on the BADL (Barthel scale) and on instrumental activities (Lawton and Brody scale), degree of cognitive deterioration (Pfeiffer's test), and time since first inclusion in the supplied service of immobile patient care and related co-morbidity.

#### *Caregiver variables*

• Socio-demographic: sex, age, marital status, educational level, and socioeconomic level.

• Kinship with the care-receiver and number of household members.

• Time (years) since first functioning as a caregiver and presence or absence of formal support (defined as professional or institutional received help).

• EuroQol-5D [21]: EQ-5D Dimensions, EQ-5D Index (index of preference-based measurements or utilities).

• Presence or absence of care problems related to the role as caregiver (nursing diagnosis NANDA-I), degree of caregiver effort (Zarit's scale), signs of depression (Yesavage's test), degree of anxiety (Goldberg's scale), and perception of family function (Apgar family test).

### Data collection method

Information will be collected via a clinical interview and data will be recorded in an electronic data collection notebook specifically designed for this trial. Variables will be collected during 4 visits: at the beginning of the trial, at the end of the intervention, and at 6 and 12 months after the intervention. The timing for the data collection is shown in Table 1.

**Table 1:** Procedures to be followed and information to be recorded at each patient’s visit

	
Step 1	Recruitment of caregivers
	- Presentation/information about the trial
	- Invitation to participate
	- Checking inclusion/exclusion criteria
	- Signing of informed consent
	- Collection of sociodemographic variables (dependent patient and caregiver)
Step 2	Primary health care center randomization
Step 3	Base-line visit
	Collection of variables on:
	- Dependent Patient: degree of cognitive deterioration and dependency level.
	- Evaluation of formal support.
	- Caregiver: self-perceived quality of life, degree of burden, evaluation of anxiety/depression degree, Apgar family test, and nursing diagnosis.
Step 4	Intervention period (duration of 6 months)
	**Intervention group**
	Standard care + CuidaCare intervention
	**Control group**
	Standard care
Step 5	Follow-up (0 months after intervention)
	Collection of variables on:
	- Dependent Patient: degree of cognitive deterioration and dependency level.
	- Evaluation of formal support.
	- Caregiver: Self-perceived quality of life, degree of burden, evaluation of anxiety/depression degree, Apgar family test, and nursing diagnosis.
Step 6	Follow-up (6 months after intervention)
	Collection of variables on:
	- Dependent patient: degree of cognitive deterioration and dependency level.
	- Evaluation of formal support.
	- Caregiver: Self-perceived quality of life, degree of burden, evaluation of anxiety/depression degree, Apgar family test, and nursing diagnosis.
	**Intervention group**
	Standard care + reinforcement of CuidaCare intervention
	**Control group**
	Standard care
Step 7	Follow-up (12 months after intervention). End of trial.
	Collection of variables on:
	- Dependent Patient: degree of cognitive deterioration and dependency level.
	- Evaluation of formal support.
	- Caregiver: self-perceived quality of life, degree of burden, evaluation of anxiety/depression degree, Apgar family test, and nursing diagnosis.

### Losses to follow-up

The number of caregivers who decline to participate in the trial will be recorded according to the requirements of the CONSORT declaration [31]; data on their age and sex will be collected. Similarly, losses to follow-up and abandonments, as well as the relevant causes, will be recorded during the study. With the intention of minimizing such losses to follow-up, caregivers will be contacted by phone to schedule the visits.

### Withdrawal criteria

Main caregivers who, once recruited, sicken of a severe pathology.

Main caregivers of dependent patients who are hospitalized for more than 3 months over the trial period.

### Analysis

1. Descriptive analysis of each variable with its corresponding 95% confidence interval (CI). Tests for normality. Description of the profile of patients who abandon the study, and their reason for withdrawal.

2. Comparison of the group at the beginning of the trial for response variables, descriptive variables, and prediction factors. Bivariate, statistical, and other tests appropriate for the type of variable (qualitative or quantitative) will be used.

3. Analysis of primary outcome at the end of the intervention (0 month of follow-up period), and at 6 and 12 months after the intervention. The average change in EQ-5D VAS value before and after the intervention will be compared between the two groups by using parametric and/or non-parametric tests and the CI of the difference will be calculated. A mixed-effects regression model will be used to adjust by prognostic factors. Confounding factors or factors that may alter the recorded effect will be taken into account in this analysis.

4. Analysis of secondary outcome. For each of the secondary response variables, the variable outcomes will be compared based on the assigned group by calculating the difference of means or proportions of each variable, as well as CIs.

All statistical tests will be performed as intention-to-treat. The last and baseline observations carried forward will be used for missing data. Significance will be set at p < 0.05.

Researchers in charge of performing the analysis will not know which intervention has been assigned to each of the included patients.

### Ethical considerations

This trial has been approved by the Ethics Board of Clinical Research of Hospital Príncipe de Asturias (date May 26, 2011, OE 09/2011 CP 11/02132) and positively evaluated by the Comisión Central de Investigación de la Gerencia de Atención Primaria de Madrid (report dated March 23, 2011). The trial will fulfill the basic principles of the Helsinki Declaration (2008), Good Clinical Practice standards, and current Spanish legislation (Real Decreto 223/2004).

The researcher will properly inform the subjects who participate in the trial and written, informed consent will be requested, signed, and dated. Participants will be given oral and written, complete, and adequate information about the nature, purpose, and possible risks and benefits of their participation in the trial.

## Discussion

Systematic reviews of studies [14-17] evaluating the effectiveness of interventions and caregiving strategies for the caregiver show methodological limitations in their conclusions, mainly related to insufficient sample sizes, high losses to follow-up of subjects, and follow-up period duration. They also reveal the variability of the interventions and outcome variables employed.

The CuidaCare intervention is a personal intervention, aimed at both the caregiver and the dependent patient, and based on coping techniques.

The design selected for this project is the best possible given the intervention, while seeking to avoid possible contamination effects among centers. The number of clusters is sufficient for the randomization to balance potentially confounding factors among themselves and we consider modelization techniques which take into account the intracluster correlation.

Given the nature of the intervention, it cannot be masked. However, we have designed a blind evaluation of the outcome.

Subjects will be recruited for the study by their own nurses. This increases variability, which we attempt to minimize by training all participating nurses on the one hand, and by creating a research protocol and implementing an electronic data collection notebook on the other hand.

Of note, the fact that nurses recruit their own patients increases implementation in standard practice.

Participation of nurses is voluntary. We are aware that a bias is introduced, but they are motivated and engaged professionals who will facilitate the development of innovative interventions such as CuidaCare.

Despite loses to follow-up in primary care being minimal as a result of the user's proximity to the system and the possibilities the health professional has to contact relatives and friends, we have estimated a high percentage in the trial due to the high morbidity-mortality of dependent patients, which will force the caregiver to abandon the trial due to the patient's hospitalization or death.

The design of the trial is pragmatic, which will allow evaluating not only its effectiveness, but also its applicability in the context of primary care.

We consider that a structured, standardized intervention can improve the quality of life of older caregivers. Detailed information about the intervention, informal care, and dependency degree of the relative receiving care can contribute to understanding what types of strategies result in improvements to the quality of life of the caregiver, and to pay attention to relevant factors which allow for designing more specific and homogeneous care plans for this population.

Currently, studies on the effectiveness of nursing interventions are very limited in this field. This project can contribute to opening new research lines on nursing approaches to care problems of informal caregivers in primary care, help compare the effectiveness among different nursing interventions, and their associated cost-effectiveness analysis.

## Abbreviations

BADL: Basic activities of daily living; CI: Confidence interval; EQ-5D: EuroQol-5D; HRQL: Health-related quality of life; PHCC: Primary health care center; NANDA-I: North American Nursing Diagnosis Association-International; NIC: Nursing interventions classification; NOC: Nursing outcomes classification; VAS: Visual analogue scale.

## Competing interests

All authors declare that they have no competing interests.

## Authors’ contributions

MRB, EEM, and CFA conceived the study. MRB, EEM, AVP, ICG, and TSC participated in the design of the study. PGS, NHV, MMM, LDP, MFR, ARA, JSB, CPH, EEM, and MRB collaborated in the coordination of the study and the design of the CuidaCare intervention. AVP, MRB, EEM, PGB, and GCC collaborated in the bibliographical search. YRF has contributed in designing the analysis. MRB, PGB, GCC, ICG, TSC, and EEM wrote the manuscript. All authors critically evaluated the content of this article until the final version was approved. All authors read and approved the final manuscript.

## Authors’ information

Milagros Rico-Blázquez, Esperanza Escortell-Mayor, Isabel del-Cura-González, Teresa Sanz-Cuesta, Sonia Soto-Diaz and Yolanda Ramallo-Fariña: Network Services Research Restroom Diseases (REDISSEC).

## Pre-publication history

The pre-publication history for this paper can be accessed here:

http://www.biomedcentral.com/1472-6955/13/2/prepub

## Supplementary Material

Additional file 1 **Cluster consort check list.** Review cluster consort check list. Click here for file
